# Early Psychiatric Impact of COVID-19 Pandemic on the General Population and Healthcare Workers in Italy: A Preliminary Study

**DOI:** 10.3389/fpsyt.2020.561345

**Published:** 2020-12-22

**Authors:** Benedetta Demartini, Veronica Nisticò, Armando D'Agostino, Alberto Priori, Orsola Gambini

**Affiliations:** ^1^Dipartimento di Scienze della Salute, Università degli Studi di Milano, Milano, Italy; ^2^Unità di Psichiatria II, Presidio San Paolo, ASST Santi Paolo e Carlo, Milano, Italy; ^3^“Aldo Ravelli” Research Center for Neurotechnology and Experimental Brain Therapeutics, Università degli Studi di Milano, Milano, Italy

**Keywords:** COVID-19, SARS-CoV-2, stress, anxiety, depression, PTSD, sleep, healthcare workers

## Abstract

**Introduction:** Since February 2020, the outbreak of COVID-19 spread to several countries worldwide, including Italy. In this study, we aimed to assess the psychopathological impact of the pandemic across the general population of Lombardy, the most affected Italian region, and to compare the prevalence of psychiatric symptoms between the general public and healthcare workers.

**Methods:** Four hundred and thirty-two participants completed an online survey including: the Depression, Anxiety and Stress Scale−21 items (DASS-21), the Impact of Event Scale—Revised (IES-R) and the Pittsburgh Sleep Quality Index (PQSI). Healthcare workers were also asked to complete the Maslach Burnout Inventory (MBI).

**Results:** At the DASS-21, 33.3% of the responders presented pathological levels of stress, 25.5% of anxiety, and 35.9% of depression. At the IES-R, 13.9% appeared at risk of developing Post-Traumatic Stress Disorder (PTSD). At the PSQI, 57.6% presented sleep disturbances. Female gender and younger age predicted higher scores of distress. Healthcare workers presented higher levels of psychiatric symptoms than the general public. Moreover, working in contact with COVID-19 patients predicted higher scores at the IES-R subscale Intrusion.

**Conclusion:** Our results showed that about a third of our sample presented symptoms of stress, anxiety, and depression during the first month of the COVID-19 pandemic outbreak in Lombardy; more than half of the responders presented sleep disturbances, and 13% appeared at risk of PTSD. Italian authorities should develop specific strategies to guarantee psychological support to the population of Lombardy, with particular attention to women, young people, and healthcare workers exposed to COVID-19 patients.

## Highlights

- We assessed psychiatric symptoms in Lombardy (Italy) during COVID-19 pandemic.
- A third of our sample presented symptoms of stress, anxiety, and depression.
- 13.9% appeared at risk of developing PTSD and 57.6% presented sleep disturbances.
- Healthcare workers presented higher levels of psychiatric symptoms.
- Working with COVID-19 patients predicted more Intrusion-type symptoms.

## Introduction

In December 2019, the city of Wuhan in China experienced an outbreak of atypical pneumonia caused by a novel betacoronavirus, named severe acute respiratory syndrome coronavirus 2 (SARS-CoV-2). Since February 2020, the outbreak rapidly spread to several countries worldwide. The World Health Organization officially declared coronavirus disease 2019 (COVID-19) a pandemic on March 11th 2020. At the moment of writing, Italy is one of the most affected countries, with 201,505 total cases and 27,359 deaths. With 74,348 total cases and 13,575 deaths, Lombardy is arguably the most severely stricken Italian region. The rate of transmissibility suggested by the COVID-19 reproductive number has been estimated at around 4 (1), indicating that every infected person might transmit the infection up to 4 people. In order to limit the outbreak, Italian authorities ordered a strict quarantine at the beginning of March, along with a complete lockdown of the country. The COVID-19 outbreak and subsequent measures might represent two different, albeit interconnected, risk factors for the development of psychiatric symptoms in the general population and in the subpopulation of healthcare workers who are directly involved in the management and treatment of COVID-19 patients (2). Two recent Chinese studies reported psychological distress, insomnia, anxiety, and depressive symptoms in the general public and in healthcare workers during the outbreak (3, 4). At the time of writing, only one study assessed the emotional impact of COVID-19 in the Italian general public and reported psychological distress symptoms during the early phase of the COVID-19 outbreak in a vast proportion of patients (5). However, no available studies assessed the prevalence of specific psychiatric symptoms, such as depressive, and anxious symptoms, in the Italian population. Moreover, no studies have been specifically conducted on a sample of Italian healthcare workers.

The main aim of the present study was to evaluate the prevalence of specific psychiatric symptoms (stress, anxiety, depression, sleeping disturbances) across the general public of Lombardy during the first month of COVID-19 outbreak in Italy. We also assessed the same symptoms, along with burn-out level, in a specific cohort of healthcare workers. Finally, in both populations, we aimed to identify potential risk and protective factors contributing to the development of these symptoms. This might help Italian authorities to strategically plan the promotion of mental well-being.

## Methods

This study is a cross-sectional survey, using an anonymous online questionnaire. A snowball sampling strategy was used to recruit a sample from the general public and one from healthcare workers. Data collection took place between 24th March and 31st March 2020. All participants signed an online written informed consent form before completing the questionnaire. The study was approved by the local Ethics Committee. Through the online questionnaire, demographic information was collected including age, gender, education level, and employment status; moreover, healthcare workers were requested to specify whether or not they were working in direct contact with COVID-19 patients and since how many days.

The survey included the following questionnaires.

The Depression, Anxiety, and Stress Scale−21 items (DASS-21), a strongly validated self-report questionnaire, assessing depressive, and anxiety symptoms (6). A Total Score was calculated as an index of general distress; moreover, the following three subscale scores were calculated: (i) Stress, averaging items 1, 6, 8, 11, 12, 14, 18; (ii) Anxiety, averaging items 2, 4, 7, 9, 15, 19, 20; (iii) Depression, averaging items 3, 5, 10, 13, 16, 17, 21. According to each subscale score, participants were labeled on a severity scale. Specifically, the subscale Stress score was divided into 0–7 (normal), 8–9 (mild), 10–12 (moderate), 13–16 (severe) and ≥17 (extremely severe); the subscale Anxiety score was divided into 0–3 (normal), 4–5 (mild), 6–7 (moderate), 8–9 (severe), and ≥10 (extremely severe); the subscale Depression score was divided into 0–4 (normal), 5–6 (mild), 7–10 (moderate), 11–13 (severe), and ≥14 (extremely severe). Pathological levels of either stress, anxiety, or depression were identified for participants who fell in the category of “mild” or above (7, 8).

The Impact of Event Scale-Revised (IES-R), a 22-item self-report scale that assesses subjective distress caused by traumatic events (9). The IES-R Total Score, obtained by summing the answers to each item, was divided into 0–23 (normal), 24–32 (mild psychological impact), 33–36 (moderate psychological impact), and >37 (severe psychological impact). Although the IES-R is not used to diagnose Post Traumatic Stress Disorder (PTSD), a cut-off score of 33 has been previously considered to define patients at risk of PTSD (10); moreover, three subscales were calculated, providing an indication of the level of distress experienced: (i) Intrusion (averaging the responses of items 1, 2, 3, 6, 9, 14, 16, 20); (ii) Avoidance (averaging the responses of items 5, 7, 8, 11, 12, 13, 17, 22); (iii) Hyperarousal (averaging the responses of items 4, 10, 15, 18, 19, 21).

The Pittsburgh Sleep Quality Index (PSQI) (11), investigating the quality of sleep in the month before the assessment. Seven scales were calculated following the authors' instruction: (i) Subjective Sleep Quality; (ii) Sleep Latency; (iii) Sleep Duration; (iv) Habitual Sleep Efficiency; (v) Sleep Disturbances; (vi) Use of Sleeping Medications; (vii) Daytime Dysfunction; a Total Score was calculated summing the scores of the seven subscales. Participants scoring equal or above 5 at the Total Score were considered “bad sleepers.”

Finally, healthcare workers were also requested to complete the Maslach Burnout Inventory (MBI), a validated self-report measure of burnout. Burnout is defined by the ICD-11 as “a syndrome resulting from chronic workplace stress that has not been successfully managed” (12). The MBI provides an index for three aspects of burnout: Emotional Exhaustion (summing items 1, 2, 3, 6, 8, 14, 16, 20); Depersonalization (summing items 5, 10, 11, 15, 22); and Personal Accomplishment (summing items 4, 7, 9, 12, 17, 18, 19, 21). Even in this case, according to the score at each subscale, participants were labeled on a severity scale. Specifically, the subscale Emotional Exhaustion was divided into 0–18 (low), 19–26 (moderate), ≥27 (high); Depersonalization was divided into 0–5 (low), 6–9 (moderate), ≥10 (high); Personal Accomplishment was divided into 0–33 (high), 34–39 (moderate), ≥ 40 (low) (13).

Participants showing high levels of distress were contacted and encouraged to seek for psychological counseling.

### Statistical Analysis

Statistical analysis was performed using SPSS version 26 (Statistical Package for Social Science). The significance level was set at α = 0.05, and all tests were 2-tailed.

A series of dichotomic variables was created according to the results of the psychometric questionnaires: participants who scored above the aforementioned cut-offs for each scale / subscale were labeled Clinical (1), and participants scoring below were labeled Not Clinical (0).

First, descriptive statistics were calculated for sociodemographic characteristics and for scales score.

Second, two categorical variables were created: (i) Group, dividing healthcare workers (HW) and general public (GP); (ii) COVID-19, dividing healthcare workers in contact (CHW) and not in contact (NCHW) with COVID-19 patients.

Mann-Whitney U test was run to assess differences amongst the groups (HW vs. GP, and CHW vs. NCHW) for the demographic variables and for the results at the questionnaires. Categorical variables were analyzed via Pearson Chi Square (χ^2^) test. Finally, we used multiple linear regression analysis to calculate whether sociodemographic characteristics predicted the presence of psychiatric symptoms in three groups: (i) the whole sample; (ii) healthcare workers only; (iii) healthcare workers exposed to COVID-19 patients only.

Analytical code is available in Supplementary Material 1.

## Results

### Demographic Features

A total of 432 valid questionnaires was retrieved. All responders were Italian and were living in Lombardy at the time of testing. Three–hundred and eleven responders (72%) were female and two (0.5%) preferred not to declare their gender. The mean age of the total sample was 35.9 (± 12.1) years old, and mean education was 16.8 (± 2.8) years. Sixty-six participants (15.3%) were students, 357 were employed (82.6%), one was unemployed (0.2%), and eight were retired (1.9%). In our sample, 123 (28.5%) were healthcare workers and 49 of them (39.9%) were working directly in contact with patients affected by COVID-19.

### Psychopathological Assessment

According to the DASS-21 subscales, 144 responders (33.3%) had pathological levels of stress, 110 (25.5%) of anxiety, and 155 (35.9%) of depression. At the IES-R Total Score, 60 participants (13.9%) appeared to be at risk of PTSD. Finally, 249 (57.6%) were found to be “bad sleepers” at the PSQI Total Score.

Within the HW group only, 59 responders (48%) presented pathological levels of stress, 47 (38.2%) of anxiety, and 51 (41.5%) of depression; 23 (18.7%) appeared to be at risk of PTSD according to the IES-R Total Score and 88 (71.5%) fell in the “bad sleepers” category at the PSQI Total Score. According to the MBI subscales, 47 (38.2%) healthcare workers presented high levels of emotional exhaustion, 49 (39.8%) of depersonalization, and 59 (48%) low levels of personal accomplishment.

Finally, within CHW only, 28 responders (57.1%) presented pathological levels of stress, 23 (46.9%) of anxiety, and 25 (51%) of depression; 11 (22.4%) appeared to be at risk of PTSD according to the IES-R Total Score and 35 (71.4%) fell in the “bad sleepers” category at the PSQI Total Score. According to the MBI subscales, 28 (57.1%) of healthcare workers presented high levels of emotional exhaustion, 24 (49%) high rates of depersonalization, and 21 (42.9%) low levels of personal accomplishment.

Further details are available in Supplementary Material 2.

### Healthcare Workers (HW) vs. General Public (GP)

Groups were balanced for gender [χ (2) = 4.838, *p* = 0.089] and age [U (432) = 21,249, *p* = 0.055]; HW had a higher level of education than GP [U (432) = 24,952, *p* < 0.001].

Significant differences emerged between the two groups at the DASS-21 Total Score [U (432) = 24,388, *p* < 0.001] and at all the DASS-21 subscales: (i) Stress [U (432) = 24,483.5, *p* < 0.001]; (ii) Anxiety [U (432) = 25,230.5, *p* < 0.001]; (iii) Depression [U (432) = 21,619.5, *p* = 0.025], all with HW scoring higher than GP.

Healthcare workers scored higher also at the IES-R Total Score [U (432) = 22,408.5, *p* = 0.004] and at the IES subscales Intrusion [U (432) = 24,098, *p* < 0.001], and Hyperarousal [U (432) = 21,519, *p* = 0.031]. No difference was found at the IES subscale Avoidance (*p* > 0.05).

Finally, significant differences were found at the PSQI Total Score [U (432) = 23,846.5, *p* < 0.001] and at the following PSQI subscales: (i) Subjective Sleep Quality [U (432) = 22,546, *p* < 0.001]; (ii) Sleep Duration [U (432) = 25,423.5, *p* < 0.001]; (iii) Sleep Efficiency [U (431) = 21,308, *p* = 0.011]; (iv) Daytime Dysfunction [U (432) = 21,644.5, *p* = 0.007], all with HW scoring higher than GP (therefore having a worse sleep quality).

See Table 1 for further details.

**Table 1:** Sociodemographic and psychometric assessment.

	**Whole sample**			**Healthcare workers**		
	**GP (*N* = 309)**	**HW (*N* = 123)**	***p***	**NCHW (*N* = 74)**	**CHW (*N* = 49)**	***p***
Age, years, mean (SD)	35.91 (13)	36 (9.2)	0.055	35.7 (9.4)	36.5 (9.2)	0.555
Gender	94 M, 214 F, 1 Undeclared	25 M, 97 F, 1 Undeclared	0.089	13 M, 61 F	12 M, 36 F, 1 Undeclared	0.286
Years of education, mean (SD)	16.3 (3)	18 (1.9)	** <0.001**	18 (1.9)	18 (1.8)	0.967
Time directly in contact with COVID-19 patients, days, mean (SD)	N/A	N/A	N/A	N/A	17.9 days (10.2)	N/A
DASS-21 total Score, score, mean (SD)	11.5 (9.3)	15.4 (9.2)	** <0.001**	14.9 (9.9)	16.1 (8.2)	0.222
DASS-21 stress, score, mean (SD)	5.74 (3.9)	7.7 (4)	** <0,001**	7.4 (4.1)	8.2 (3.9)	0.236
DASS-21 anxiety, score, mean (SD)	2 (3)	3.4 (3.2)	** <0.001**	3.3 (3.4)	3.5 (3)	0.485
DASS-21 depression, score, mean (SD)	3.8 (3.7)	4.3 (3.3)	**0.025**	4.3 (3.5)	4.4 (3)	0.464
IES-R total score, score, mean (SD)	17.4 (12.8)	21.2 (12.1)	**0.004**	20.1 (13.7)	22.9 (14.8)	0.322
IES-R avoidance, score, mean (SD)	0.8 (0.6)	0.9 (0.6)	0.633	0.8 (0.6)	0.9 (0.6)	0.811
IES-R intrusion, score, mean (SD)	0.8 (0.7)	1 (0.8)	** <0.001**	1 (0.7)	1.3 (0.8)	0.058
IES-R hyperarousal, score, mean (SD)	0.8 (0.7)	1 (0.8)	**0.031**	0.9 (0.8)	1 (0.8)	0.665
PSQI total score, score, mean (SD)	5.1 (2.7)	6.8 (3.6)	** <0.001**	6.7 (3.7)	7 (3.6)	0.584
PSQI subjective sleep quality, score, mean (SD)	1 (0.6)	1.3 (0.7)	** <0.001**	1.2 (0.7)	1.3 (0.7)	0.451
PSQI sleep latency, score, mean (SD)	1 (0.9)	1.2 (1)	0.149	1.2 (1)	1.2 (1)	0.982
PSQI Sleep Duration,score, mean (SD)	0.7 (0.8)	1.3 (1)	** <0.001**	1.3 (1)	1.4 (1)	0.478
PSQI habitual sleep efficiency, score, mean (SD)	0.4 (0.7)	0.7 (1)	**0.011**	0.5 (0.9)	0.9 (1)	**0.012**
PSQI sleep disturbances, score, mean (SD)	1 (0.5)	1.1 (0.5)	0.211	1 (0.5)	1 (0.6)	0.644
PSQI use of sleeping medications, score, mean (SD)	0.3 (0.8)	0.4 (0.9)	0.165	0.5 (1)	0.3 (0.7)	0.298
PSQI daytime dysfunction, score, mean (SD)	0.7 (0.5)	0.9 (0.6)	**0.007**	0.9 (0.6)	0.9 (0.4)	0.702
MBI emotional exhaustion, score, mean (SD)	N/A	25.1 (12.1)	N/A	22 (9.7)	29.1 (13.7)	**0.008**
MBI depersonalization, score, mean (SD)	N/A	10.3 (5.2)	N/A	9.6 (4.7)	11.3 (5.8)	0.09
MBI personal achievement, score, mean (SD)	N/A	32.2 (9.4)	N/A	30.7 (9.4)	34.1 (9.2)	**0.051**

*CHW, healthcare workers directly in contact with COVID-19 patients; DASS-21, Depression, Anxiety, and Stress Scale−21 items; GP, General Public; HW, healthcare Workers; IES-R, Impact of Event Scale-Revised; MBI, Maslach Burnout Inventory; N/A, Not Applicable; NCHW, healthcare workers not directly in contact with COVID-19 patients; PSQI, Pittsburgh Sleep Quality Index. Bold values mean p < 0.05*.

### COVID-19 Healthcare Workers (CHW) vs. Non-COVID-19 Healthcare Workers (NCHW)

Groups were balanced for gender [χ (2) = 2.506, *p* = 0.286], age [U (123) = 1,927, *p* = 0.555], and years of education [U (123) = 1,807.5, *p* = 0.967]

No group differences were found at the DASS-21 Total Score, nor at the DASS-21 subscales. Similarly, no differences emerged at the IES-R Total Score and subscales. A trend toward significance appeared for the IES-R subscale Intrusion [U (123) = 2,180, *p* = 0.058], with CHW scoring higher than NCHW. CHW scored higher than NCHW at the PSQI subscale Habitual Sleep Efficiency [U (122) = 2,210.5, *p* = 0.012] and at the MBI subscale Emotional Exhaustion [U (114) = 2,058.5, *p* = 0.008]; finally, a trend toward significance emerged at the MBI subscale Personal Accomplishment [U (114) = 1,932.5, *p* = 0.051], with CHW scoring higher than NCHW.

See Table 1 for further details.

### Regression Analysis

#### Regression Analysis in the Whole Sample

The female gender was a predictor of the presence of psychiatric symptoms in terms of stress, anxiety, PTSD-like symptoms and sleep disturbances. In particular, the variable Gender was a predictor of: (i) the DASS-21 Total Score (*p* = 0.023) and the DASS-21 subscales Stress (*p* = 0.03) and Anxiety (*p* = 0.003); (ii) the IES-R Total Score (*p* < 0.001) and all the IES-R subscales [Avoidance (*p* = 0.032), Intrusion (*p* < 0.001), and Hyperarousal (*p* = 0.001)]; (iii) the PSQI subscales Subjective Sleep Quality (*p* = 0.003), Sleep Latency (*p* = 0.02), and Sleep Disturbances (*p* = 0.001).

Symptoms of stress, anxiety and PTSD-like symptoms increased as age lowered; on the contrary, sleep disturbances increased with age. In particular, the variable Age was a predictor of: (i) the DASS-21 Total Score (*p* = 0.002) and the DASS-21 subscales Stress (*p* < 0.001) and Anxiety (*b* = −0.029; *t* = −2.39; *p* = 0.017); (ii) the IES-R subscales Avoidance (*p* = 0.010) and Hyperarousal (p = 0.025); (iii) the PSQI Total Score (*p* = 0.002) and its subscales Subjective Sleep Quality (*p* = 0.003), Sleep Duration (*p* < 0.001), and Use of Sleeping Medication (*p* < 0.001).

Being a healthcare worker was a predictor of the presence of psychiatric symptoms in terms of stress, anxiety, PTSD-like symptoms and sleep disturbances. In particular, being a healthcare worker was a predictor of: (i) the DASS-21 Total Score (*p* < 0.001) and its subscales Stress (*p* < 0.001) and Anxiety (*p* < 0.001); (ii) the IES-R Total Score (*p* = 0.032) and its subscale Intrusion (*p* < 0.001); (iii) the PSQI Total Score (*p* < 0.001) and its subscales Sleep Duration (*p* < 0.001), Habitual Sleep Efficiency (*p* = 0.004) and Daytime Dysfunction (*p* = 0.02).

None of the psychometric variables was predicted by responders' educational level.

#### Regression Analysis Within the Healthcare Workers Group

Again, the female gender was a predictor of the presence of psychiatric symptoms in terms of stress, anxiety, PTSD-like symptoms, sleep disturbances and burnout. In particular, the variable Gender was a predictor of: (i) the DASS-21 Total Score (*p* = 0.008) and its subscales Stress (*p* = 0.008) and Anxiety (*p* = 0.001); (ii) IES-R Total Score (p = 0.024) and its subscales Intrusion (*p* = 0.033) and Hyperarousal (*p* = 0.01); (iii) the PSQI subscales Subjective Sleep Quality (*p* = 0.014) and Sleep Disturbances (*p* = 0.023); (iv) the MBI subscale Emotional Exhaustion (*p* = 0.044).

Sleep disturbances were higher at a higher age. In fact, the variable Age was a predictor of the PSQI Total Score (*p* = 0.004) and its subscales Sleep Duration (*p* = 0.004), Habitual Sleep Efficiency (*p* = 0.021), and Use of Sleeping Medication (*p* = 0.006).

Working directly in contact with COVID-19 patients was a predictor of the IES-R subscale Intrusion (p = 0.021) and the MBI subscale Emotional Exhaustion (*b* = 7.245, *t* = 3.337, *p* = 0.001), both with CHW presenting more symptoms of intrusion and emotional exhaustion than NCHW.

#### Regression Analysis Within the COVID-19 Healthcare Workers Group

Gender was a predictor of the DASS-21 subscale Anxiety (*p* = 0.01): in particular, female gender predicted the presence of anxiety.

Age was a predictor of the PSQI subscale Use of Sleeping Medication (*p* < 0.001), with use of medication increasing with age.

The time (measured in days) spent directly in contact with COVID-19 patients was a predictor of the PSQI subscale Sleep Disturbances (*p* = 0.005): sleep disturbances increased with the time spent with COVID-19 patients.

Further details, including statistical indexes, are available in Supplementary Material 3.

## Discussion

In this study, we aimed to evaluate the prevalence of specific psychiatric symptoms across the general population and within a specific subsample of healthcare workers in the region of Lombardy, during the first month of the COVID-19 outbreak in Italy. Although we acknowledge that our findings might have been biased by the relatively small sample size and the majority of women in the sample, we strongly believe that our preliminary data should be taken into account as a first evidence of the psychological distress experienced by the population of Lombardy during the COVID-19 outbreak and the consequent lockdown measures. In particular, our data revealed an estimated prevalence of 25.5, 35.9, and 33.3% for symptoms of anxiety, depression, and stress, respectively. Moreover, 13.9% of the whole sample appeared to be at risk of developing PTSD. Our results are in line with those obtained both in the Chinese population, at the epicenter of the pandemic (3, 4), and in the general Italian population (5). In the first few weeks of the outbreak, Moccia et al. (5) reported that 38% of the Italian general population presented mild to severe psychological distress, which was related to specific temperament characteristics (cyclothymic, depressive, anxious) and adult attachment style. Here we expanded these findings by assessing specific psychiatric symptoms (anxiety, depression, stress, PTSD-like symptoms, and sleep disturbances); furthermore, we selected the population of Lombardy, the most affected Italian region with the largest number of infected people and deaths, accounting for almost half of all cases in Italy. Although the Italian population endured several traumatic events in the last decades (e.g., the series of earthquakes and tremors hitting central Italy since 2009), the entity of the current pandemic and measures taken by Italian authorities to contain the outbreak are unprecedented in the country's modern history. Our study provides an early insight on the psychopathological impact of this phenomenon in a large sample of individuals. Moreover, this is the first study to investigate participants' quality of sleep and over half the sample (57.6%) was found to be a “bad sleeper” during the first month of the outbreak. Insomnia has previously been reported in several studies on mental health during quarantine, albeit with lower prevalence: Lee et al. (14) reported insomnia in 34.2% of residents at Amoy Gardens, the first officially recognized site of the SARS, 2003 community outbreak in Hong Kong; similar results were found the same year for inpatients with SARS in Canada (15) and for contacts of patients with Ebola in Senegal in 2014 (16). However, this is the first study to specifically assess sleep disturbances during the COVID-19 pandemic, and the high prevalence of bad sleepers might depend on the wide range of sleep disturbances assessed by the scale employed. Indeed, the PSQI is not restricted to insomnia but includes nightmares, feeling too hot or too cold while sleeping and sleep–related daytime disturbances.

We also identified predictors of high stress and psychiatric symptoms to provide indications for early psychological or psychiatric interventions. Our findings suggest that female gender represents a risk factor for the development of stress, anxiety, and sleep disturbances, together with PTSD symptoms Intrusion-type, Avoidance-type, and Hyperarousal-type. This finding, which is in line with studies conducted both in the context of previous epidemics (17) and in the context of the COVID-19 pandemic in China (4), suggests early interventions should be tailored for women. On one hand, higher age was found to be a protective factor for the development of stress, anxiety, depression and PTSD-like symptoms in our sample. On the other, it was found to predict a globally worse quality of sleep and an increased tendency to use sleeping medications. Taken together, these findings suggest that earlier interventions should be focused on younger individuals to address emotional distress and older ones for sleep disturbances.

In this study, we also compared the prevalence of symptoms between the general public and a subsample of healthcare workers in Lombardy. Similar studies have been conducted in the Chinese population during the outbreak of the COVID-19 pandemic (3, 18, 19) and in the context of other epidemics in the past, such as SARS in Canada in 2003 (10). Previous studies consistently reported a higher risk of developing psychiatric distress and sleep disturbances in HW, when compared to the general public [a review, see Brooks et al. (20)]. To the best of our knowledge, this is the first study to investigate the psychopathological impact of the COVID-19 pandemic on healthcare workers in Italy. Our findings are in line with the aforementioned studies, showing higher levels of stress, anxiety and depression than the general population, together with more PTSD-like symptoms (in particular, intrusion-type and hyperarousal-type) and sleep disturbances. Female gender was confirmed to be a predictor of (i) higher levels of stress and anxiety, (ii) PTSD symptoms of Intrusion-type and Hyperarousal-type, and (iii) sleep disturbances in this sample. Although any attempt to frame these preliminary finding should be considered speculative, the gender inequality issues that dominate Italian society might have a role. Recent research has shown women healthcare workers to be discriminated in terms of salary and career progression (21). Furthermore, Italian welfare and social policy regimes often fail to address the needs of working women, who might experience a higher burden of distress during an unprecedented emergency that generates existential and collective uncertainty.

Furthermore, working directly in contact with COVID-19 patients appeared to be a predictor of the levels of Intrusion-type PTSD symptoms, regardless of the sociodemographic characteristics of healthcare workers, such as age and gender. The IES-R subscale Intrusion assesses the presence of repeated thoughts about the traumatic event (e.g., “Other things kept making me think about it.” “Pictures about it popped into my mind.” and “I had dreams about it.”). Reynolds et al. (10) used the same instrument to assess the prevalence of PTSD in a group of healthcare workers operating against SARS in Canada in 2003; they found that the average score of healthcare workers at the Intrusion subscale was 0.7 (± 0.9), similar to the one found in our sample of healthcare workers (1 ± 0.8). In this study, we further showed that even after controlling for sociodemographic characteristics such as age and gender, healthcare workers directly in contact with COVID-19 patients are most at risk of developing Intrusion-type PTSD symptoms. Among CHW, female gender predicted higher levels of anxiety and higher age predicted an increased use of sleeping medications. Interestingly, the time spent directly in contact with patients with COVID-19 was a predictor of the PSQI subscale Sleep Disturbances. This scale evaluates the frequency of different causes of sleep perturbation, such as: “cannot breath comfortably,” “feeling too hot/too cold,” “having bad dreams.” It seems, therefore, that sleep disturbances increased with time spent with COVID-19 patients.

Finally, burnout levels were assessed within healthcare workers and compared between CHW and NCHW. High levels of Emotional Exhaustion and Depersonalization were presented, respectively, in 38.2 and 39.8% of the healthcare workers; furthermore, 48% of HW presented low levels of Personal Accomplishment, suggesting a worse satisfaction on the workplace and a sense of inadequacy about one's ability to relate to patients. CHW presented higher levels of Emotional Exhaustion, a scale describing the feeling of having no more emotional resources to cope with the situation at work (e.g., “I felt emotionally drained from my work”). This is in line with the findings of Marjanovic et al. (22), conducted on a group of nurses coping with the epidemy of SARS in Canada: they showed that a minor contact with SARS patients, together with a greater trust in the available equipment and in the infection control initiatives, predicted lower levels of emotional exhaustion.

Besides the small sample size and the majority of women, already mentioned at the beginning of the discussion, we acknowledge the following limitations: first, the use of an online survey did not allow the researchers to time the participants while administering it, to explain the study objectives directly and to debrief the participants; second, the recruitment via snowball sampling strategy and the lack of a longitudinal follow-up might limit the generalizability of our results; third, we did not distinguish between people affected and not-affected by COVID-19, and we did not assess specific personality and psychological characteristics of our sample; thus, predictors of psychiatric symptoms could only be explored within the known sociodemographic characteristics of our sample (age, gender, years of educations).

## Conclusion

In conclusion, our results showed that about a third of our sample, recruited amongst inhabitants of Lombardy, Italy, presented psychiatric symptoms of stress, anxiety and depression during the first month of the COVID-19 pandemic outbreak; more than half of the responders to our survey presented sleep disturbances, and 13% appeared at risk of developing PTSD. Furthermore, younger age and female gender appeared to be risk factors for the development of psychiatric symptoms. These results might prove useful to Italian authorities that will strategically coordinate the promotion of mental well-being in upcoming months. Specific interventions tailored to the needs of healthcare workers, especially those directly exposed to patients with COVID-19, are also warranted.

## Data Availability Statement

The raw data supporting the conclusions of this article will be made available by the authors, without undue reservation.

## Ethics Statement

The studies involving human participants were reviewed and approved by the Ethics Committee of ASST Santi Paolo e Carlo, Milan, Italy. The patients/participants provided their written informed consent to participate in this study.

## Author Contributions

BD, VN, and OG conceived and designed the experiment. BD and VN performed data collection. VN analyzed the data, under supervision of BD and AD'A. BD, VN, and AD'A wrote the manuscript. AP and OG revised the final manuscript. All authors approved the manuscript before submission. All authors contributed to the article and approved the submitted version.

## Conflict of Interest

The authors declare that the research was conducted in the absence of any commercial or financial relationships that could be construed as a potential conflict of interest.
