# Supplementary material for: Early Psychiatric Impact of COVID-19 Pandemic on the General Population and Healthcare Workers in Italy: A Preliminary Study
Source: Front Psychiatry. 2020 Dec 22;11:561345. doi: 10.3389/fpsyt.2020.561345 (PMC7783153; doi:10.3389/fpsyt.2020.561345)
Supplement: Supplementary file 1 [file Data_Sheet_1.DOCX]

* Encoding: UTF-8.

DESCRIPTIVES VARIABLES=Age Sex Time_covid Anni_studio DASS_Stress DASS_Anxiety

DASS_Depression DASS_Tot IES_Evitamento IES_Intrusività IES_Iperarousal IES_Tot_AV IES_Tot_SUM

P_Subjective_Sleep_Quality P_Sleep_Latency P_Sleep_Duration P_Habitual_Sleep_Efficiency

P_Sleep_Disturbances P_Use_Sleeping_Medication P_Daytime_dysfunction Pittsburgh_Total

MBI_Emotional_exhaustion MBI_Depersonalization MBI_Personal_Accomplishment

/STATISTICS=MEAN STDDEV MIN MAX.

RECODE Stress (Lowest thru 7=0) (8 thru 9=1) (10 thru 12=2) (13 thru 16=3) (17 thru Highest=4) INTO

Stress_Cat.

EXECUTE.

RECODE Anxiety (Lowest thru 3=0) (4 thru 5=1) (6 thru 7=2) (8 thru 9=3) (10 thru Highest=4) INTO

Anxiety_Cat.

EXECUTE.

RECODE Depression (Lowest thru 4=0) (5 thru 6=1) (7 thru 10=2) (11 thru 13=3) (14 thru Highest=4) INTO

Depression_Cat.

EXECUTE.

RECODE Stress_Cat Anxiety_Cat Depression_Cat (0=0) (1 thru Highest=1) INTO Stress_Dic Anx_dic

Depr_dic.

EXECUTE.

RECODE IES_Tot_SUM (Lowest thru 23=0) (24 thru 32=1) (33 thru 36=3) (37 thru Highest=4) INTO

ies_Cat.

EXECUTE.

RECODE ies_Cat (Lowest thru 1=0) (2 thru Highest=1) INTO ies_dic.

EXECUTE.

RECODE Pittsburgh_Total (Lowest thru 4=0) (5 thru Highest=1) INTO PQSI_CAT.

EXECUTE.

RECODE MBI_Emotional_exhaustion (Lowest thru 18=0) (19 thru 26=1) (27 thru Highest=2) INTO EE_CAT.

EXECUTE.

RECODE MBI_Depersonalization (Lowest thru 5=0) (6 thru 9=1) (10 thru Highest=2) INTO dep_cat.

EXECUTE.

RECODE MBI_Personal_Accomplishment (Lowest thru 33=0) (34 thru 39=1) (40 thru Highest=2) INTO

PA_CAT.

EXECUTE.

FREQUENCIES VARIABLES=stress_cat anx_cat depr_Cat ies_Cat PQSI_CAT ies_dic stress_dic anx_dic

depr_dic EE_CAT dep_cat PA_CAT

/ORDER=ANALYSIS.

SORT CASES BY Group.

SPLIT FILE LAYERED BY Group.

FREQUENCIES VARIABLES=stress_cat anx_cat depr_Cat ies_Cat PQSI_CAT ies_dic stress_dic anx_dic

depr_dic EE_CAT dep_cat PA_CAT

/ORDER=ANALYSIS.

SPLIT FILE OFF.

FILTER OFF.

USE ALL.

EXECUTE.

CROSSTABS

/TABLES=Sex BY Group

/FORMAT=AVALUE TABLES

/STATISTICS=CHISQ

/CELLS=COUNT

/COUNT ROUND CELL.

EXECUTE.

*Nonparametric Tests: Independent Samples.

NPTESTS

/INDEPENDENT TEST (Age DASS_Stress DASS_Anxiety DASS_Depression IES_Evitamento IES_Intrusività

IES_Iperarousal IES_Tot_AV IES_Tot_SUM P_Subjective_Sleep_Quality P_Sleep_Latency P_Sleep_Duration

P_Habitual_Sleep_Efficiency P_Sleep_Disturbances P_Use_Sleeping_Medication P_Daytime_dysfunction

Pittsburgh_Total MBI_Emotional_exhaustion MBI_Depersonalization MBI_Personal_Accomplishment

DASS_Tot) GROUP (Group) MANN_WHITNEY

/MISSING SCOPE=ANALYSIS USERMISSING=EXCLUDE

/CRITERIA ALPHA=0.05 CILEVEL=95.

EXECUTE.

USE ALL.

COMPUTE filter_$=(Group = 1).

VARIABLE LABELS filter_$ 'Group = 1 (FILTER)'.

VALUE LABELS filter_$ 0 'Not Selected' 1 'Selected'.

FORMATS filter_$ (f1.0).

FILTER BY filter_$.

EXECUTE.

*Nonparametric Tests: Independent Samples.

NPTESTS

/INDEPENDENT TEST (Age DASS_Stress DASS_Anxiety DASS_Depression IES_Evitamento IES_Intrusività

IES_Iperarousal IES_Tot_SUM P_Subjective_Sleep_Quality P_Sleep_Latency P_Sleep_Duration

P_Habitual_Sleep_Efficiency P_Sleep_Disturbances P_Use_Sleeping_Medication P_Daytime_dysfunction

Pittsburgh_Total MBI_Emotional_exhaustion MBI_Depersonalization MBI_Personal_Accomplishment

DASS_Tot) GROUP (COVID_19) MANN_WHITNEY

/MISSING SCOPE=ANALYSIS USERMISSING=EXCLUDE

/CRITERIA ALPHA=0.05 CILEVEL=95.

EXECUTE.

FILTER OFF.

USE ALL.

EXECUTE.

REGRESSION

/MISSING LISTWISE

/STATISTICS COEFF OUTS R ANOVA

/CRITERIA=PIN(.05) POUT(.10)

/NOORIGIN

/DEPENDENT DASS_Tot

/METHOD=ENTER Sex Age Anni_studio Group.

EXECUTE.

REGRESSION

/MISSING LISTWISE

/STATISTICS COEFF OUTS R ANOVA

/CRITERIA=PIN(.05) POUT(.10)

/NOORIGIN

/DEPENDENT DASS_Stress

/METHOD=ENTER Sex Age Anni_studio Group.

EXECUTE.

REGRESSION

/MISSING LISTWISE

/STATISTICS COEFF OUTS R ANOVA

/CRITERIA=PIN(.05) POUT(.10)

/NOORIGIN

/DEPENDENT DASS_Anxiety

/METHOD=ENTER Sex Age Anni_studio Group.

EXECUTE.

REGRESSION

/MISSING LISTWISE

/STATISTICS COEFF OUTS R ANOVA

/CRITERIA=PIN(.05) POUT(.10)

/NOORIGIN

/DEPENDENT DASS_Depression

/METHOD=ENTER Sex Age Anni_studio Group.

EXECUTE.

REGRESSION

/MISSING LISTWISE

/STATISTICS COEFF OUTS R ANOVA

/CRITERIA=PIN(.05) POUT(.10)

/NOORIGIN

/DEPENDENT IES_Tot_SUM

/METHOD=ENTER Sex Age Anni_studio Group.

EXECUTE.

REGRESSION

/MISSING LISTWISE

/STATISTICS COEFF OUTS R ANOVA

/CRITERIA=PIN(.05) POUT(.10)

/NOORIGIN

/DEPENDENT IES_Evitamento

/METHOD=ENTER Sex Age Anni_studio Group.

EXECUTE.

REGRESSION

/MISSING LISTWISE

/STATISTICS COEFF OUTS R ANOVA

/CRITERIA=PIN(.05) POUT(.10)

/NOORIGIN

/DEPENDENT IES_Intrusività

/METHOD=ENTER Sex Age Anni_studio Group.

EXECUTE.

REGRESSION

/MISSING LISTWISE

/STATISTICS COEFF OUTS R ANOVA

/CRITERIA=PIN(.05) POUT(.10)

/NOORIGIN

/DEPENDENT IES_Iperarousal

/METHOD=ENTER Sex Age Anni_studio Group.

EXECUTE.

REGRESSION

/MISSING LISTWISE

/STATISTICS COEFF OUTS R ANOVA

/CRITERIA=PIN(.05) POUT(.10)

/NOORIGIN

/DEPENDENT Pittsburgh_Total

/METHOD=ENTER Sex Age Anni_studio Group.

EXECUTE.

REGRESSION

/MISSING LISTWISE

/STATISTICS COEFF OUTS R ANOVA

/CRITERIA=PIN(.05) POUT(.10)

/NOORIGIN

/DEPENDENT P_Subjective_Sleep_Quality

/METHOD=ENTER Sex Age Anni_studio Group.

EXECUTE.

REGRESSION

/MISSING LISTWISE

/STATISTICS COEFF OUTS R ANOVA

/CRITERIA=PIN(.05) POUT(.10)

/NOORIGIN

/DEPENDENT P_Sleep_Latency

/METHOD=ENTER Sex Age Anni_studio Group.

EXECUTE.

REGRESSION

/MISSING LISTWISE

/STATISTICS COEFF OUTS R ANOVA

/CRITERIA=PIN(.05) POUT(.10)

/NOORIGIN

/DEPENDENT P_Sleep_Duration

/METHOD=ENTER Sex Age Anni_studio Group.

EXECUTE.

REGRESSION

/MISSING LISTWISE

/STATISTICS COEFF OUTS R ANOVA

/CRITERIA=PIN(.05) POUT(.10)

/NOORIGIN

/DEPENDENT P_Habitual_Sleep_Efficiency

/METHOD=ENTER Sex Age Anni_studio Group.

EXECUTE.

REGRESSION

/MISSING LISTWISE

/STATISTICS COEFF OUTS R ANOVA

/CRITERIA=PIN(.05) POUT(.10)

/NOORIGIN

/DEPENDENT P_Sleep_Disturbances

/METHOD=ENTER Sex Age Anni_studio Group.

EXECUTE.

REGRESSION

/MISSING LISTWISE

/STATISTICS COEFF OUTS R ANOVA

/CRITERIA=PIN(.05) POUT(.10)

/NOORIGIN

/DEPENDENT P_Use_Sleeping_Medication

/METHOD=ENTER Sex Age Anni_studio Group.

EXECUTE.

REGRESSION

/MISSING LISTWISE

/STATISTICS COEFF OUTS R ANOVA

/CRITERIA=PIN(.05) POUT(.10)

/NOORIGIN

/DEPENDENT P_Daytime_dysfunction

/METHOD=ENTER Sex Age Anni_studio Group.

EXECUTE.

USE ALL.

COMPUTE filter_$=(Group = 1).

VARIABLE LABELS filter_$ 'Group = 1 (FILTER)'.

VALUE LABELS filter_$ 0 'Not Selected' 1 'Selected'.

FORMATS filter_$ (f1.0).

FILTER BY filter_$.

EXECUTE.

REGRESSION

/MISSING LISTWISE

/STATISTICS COEFF OUTS R ANOVA

/CRITERIA=PIN(.05) POUT(.10)

/NOORIGIN

/DEPENDENT DASS_Tot

/METHOD=ENTER Sex Age COVID_19.

EXECUTE.

REGRESSION

/MISSING LISTWISE

/STATISTICS COEFF OUTS R ANOVA

/CRITERIA=PIN(.05) POUT(.10)

/NOORIGIN

/DEPENDENT DASS_Stress

/METHOD=ENTER Sex Age COVID_19.

EXECUTE.

REGRESSION

/MISSING LISTWISE

/STATISTICS COEFF OUTS R ANOVA

/CRITERIA=PIN(.05) POUT(.10)

/NOORIGIN

/DEPENDENT DASS_Anxiety

/METHOD=ENTER Sex Age COVID_19.

EXECUTE.

REGRESSION

/MISSING LISTWISE

/STATISTICS COEFF OUTS R ANOVA

/CRITERIA=PIN(.05) POUT(.10)

/NOORIGIN

/DEPENDENT DASS_Depression

/METHOD=ENTER Sex Age COVID_19.

EXECUTE.

REGRESSION

/MISSING LISTWISE

/STATISTICS COEFF OUTS R ANOVA

/CRITERIA=PIN(.05) POUT(.10)

/NOORIGIN

/DEPENDENT IES_Tot_SUM

/METHOD=ENTER Sex Age COVID_19.

EXECUTE.

REGRESSION

/MISSING LISTWISE

/STATISTICS COEFF OUTS R ANOVA

/CRITERIA=PIN(.05) POUT(.10)

/NOORIGIN

/DEPENDENT IES_Evitamento

/METHOD=ENTER Sex Age COVID_19.

EXECUTE.

REGRESSION

/MISSING LISTWISE

/STATISTICS COEFF OUTS R ANOVA

/CRITERIA=PIN(.05) POUT(.10)

/NOORIGIN

/DEPENDENT IES_Intrusività

/METHOD=ENTER Sex Age COVID_19.

EXECUTE.

REGRESSION

/MISSING LISTWISE

/STATISTICS COEFF OUTS R ANOVA

/CRITERIA=PIN(.05) POUT(.10)

/NOORIGIN

/DEPENDENT IES_Iperarousal

/METHOD=ENTER Sex Age COVID_19.

EXECUTE.

REGRESSION

/MISSING LISTWISE

/STATISTICS COEFF OUTS R ANOVA

/CRITERIA=PIN(.05) POUT(.10)

/NOORIGIN

/DEPENDENT Pittsburgh_Total

/METHOD=ENTER Sex Age COVID_19.

EXECUTE.

REGRESSION

/MISSING LISTWISE

/STATISTICS COEFF OUTS R ANOVA

/CRITERIA=PIN(.05) POUT(.10)

/NOORIGIN

/DEPENDENT P_Subjective_Sleep_Quality

/METHOD=ENTER Sex Age COVID_19.

EXECUTE.

REGRESSION

/MISSING LISTWISE

/STATISTICS COEFF OUTS R ANOVA

/CRITERIA=PIN(.05) POUT(.10)

/NOORIGIN

/DEPENDENT P_Sleep_Latency

/METHOD=ENTER Sex Age COVID_19.

EXECUTE.

REGRESSION

/MISSING LISTWISE

/STATISTICS COEFF OUTS R ANOVA

/CRITERIA=PIN(.05) POUT(.10)

/NOORIGIN

/DEPENDENT P_Sleep_Duration

/METHOD=ENTER Sex Age COVID_19.

EXECUTE.

REGRESSION

/MISSING LISTWISE

/STATISTICS COEFF OUTS R ANOVA

/CRITERIA=PIN(.05) POUT(.10)

/NOORIGIN

/DEPENDENT P_Habitual_Sleep_Efficiency

/METHOD=ENTER Sex Age COVID_19.

EXECUTE.

REGRESSION

/MISSING LISTWISE

/STATISTICS COEFF OUTS R ANOVA

/CRITERIA=PIN(.05) POUT(.10)

/NOORIGIN

/DEPENDENT P_Sleep_Disturbances

/METHOD=ENTER Sex Age COVID_19.

EXECUTE.

REGRESSION

/MISSING LISTWISE

/STATISTICS COEFF OUTS R ANOVA

/CRITERIA=PIN(.05) POUT(.10)

/NOORIGIN

/DEPENDENT P_Use_Sleeping_Medication

/METHOD=ENTER Sex Age COVID_19.

EXECUTE.

REGRESSION

/MISSING LISTWISE

/STATISTICS COEFF OUTS R ANOVA

/CRITERIA=PIN(.05) POUT(.10)

/NOORIGIN

/DEPENDENT P_Daytime_dysfunction

/METHOD=ENTER Sex Age COVID_19.

EXECUTE.

REGRESSION

/MISSING LISTWISE

/STATISTICS COEFF OUTS R ANOVA

/CRITERIA=PIN(.05) POUT(.10)

/NOORIGIN

/DEPENDENT MBI_Emotional_exhaustion

/METHOD=ENTER Sex Age COVID_19.

EXECUTE.

REGRESSION

/MISSING LISTWISE

/STATISTICS COEFF OUTS R ANOVA

/CRITERIA=PIN(.05) POUT(.10)

/NOORIGIN

/DEPENDENT MBI_Depersonalization

/METHOD=ENTER Sex Age COVID_19.

EXECUTE.

REGRESSION

/MISSING LISTWISE

/STATISTICS COEFF OUTS R ANOVA

/CRITERIA=PIN(.05) POUT(.10)

/NOORIGIN

/DEPENDENT MBI_Personal_Accomplishment

/METHOD=ENTER Sex Age COVID_19.

EXECUTE.

FILTER OFF.

USE ALL.

EXECUTE.

USE ALL.

COMPUTE filter_$=(Group = 1 & COVID_19 = 1).

VARIABLE LABELS filter_$ 'Group = 1 & COVID_19 = 1 (FILTER)'.

VALUE LABELS filter_$ 0 'Not Selected' 1 'Selected'.

FORMATS filter_$ (f1.0).

FILTER BY filter_$.

EXECUTE.

REGRESSION

/MISSING LISTWISE

/STATISTICS COEFF OUTS R ANOVA

/CRITERIA=PIN(.05) POUT(.10)

/NOORIGIN

/DEPENDENT DASS_Tot

/METHOD=ENTER Sex Age Time_covid.

EXECUTE.

REGRESSION

/MISSING LISTWISE

/STATISTICS COEFF OUTS R ANOVA

/CRITERIA=PIN(.05) POUT(.10)

/NOORIGIN

/DEPENDENT DASS_Stress

/METHOD=ENTER Sex Age Time_covid.

EXECUTE.

REGRESSION

/MISSING LISTWISE

/STATISTICS COEFF OUTS R ANOVA

/CRITERIA=PIN(.05) POUT(.10)

/NOORIGIN

/DEPENDENT DASS_Anxiety

/METHOD=ENTER Sex Age Time_covid.

EXECUTE.

REGRESSION

/MISSING LISTWISE

/STATISTICS COEFF OUTS R ANOVA

/CRITERIA=PIN(.05) POUT(.10)

/NOORIGIN

/DEPENDENT DASS_Depression

/METHOD=ENTER Sex Age Time_covid.

EXECUTE.

REGRESSION

/MISSING LISTWISE

/STATISTICS COEFF OUTS R ANOVA

/CRITERIA=PIN(.05) POUT(.10)

/NOORIGIN

/DEPENDENT IES_Tot_SUM

/METHOD=ENTER Sex Age Time_covid.

EXECUTE.

REGRESSION

/MISSING LISTWISE

/STATISTICS COEFF OUTS R ANOVA

/CRITERIA=PIN(.05) POUT(.10)

/NOORIGIN

/DEPENDENT IES_Evitamento

/METHOD=ENTER Sex Age Time_covid.

EXECUTE.

REGRESSION

/MISSING LISTWISE

/STATISTICS COEFF OUTS R ANOVA

/CRITERIA=PIN(.05) POUT(.10)

/NOORIGIN

/DEPENDENT IES_Intrusività

/METHOD=ENTER Sex Age Time_covid.

EXECUTE.

REGRESSION

/MISSING LISTWISE

/STATISTICS COEFF OUTS R ANOVA

/CRITERIA=PIN(.05) POUT(.10)

/NOORIGIN

/DEPENDENT IES_Iperarousal

/METHOD=ENTER Sex Age Time_covid.

EXECUTE.

REGRESSION

/MISSING LISTWISE

/STATISTICS COEFF OUTS R ANOVA

/CRITERIA=PIN(.05) POUT(.10)

/NOORIGIN

/DEPENDENT Pittsburgh_Total

/METHOD=ENTER Sex Age Time_covid.

EXECUTE.

REGRESSION

/MISSING LISTWISE

/STATISTICS COEFF OUTS R ANOVA

/CRITERIA=PIN(.05) POUT(.10)

/NOORIGIN

/DEPENDENT P_Subjective_Sleep_Quality

/METHOD=ENTER Sex Age Time_covid.

EXECUTE.

REGRESSION

/MISSING LISTWISE

/STATISTICS COEFF OUTS R ANOVA

/CRITERIA=PIN(.05) POUT(.10)

/NOORIGIN

/DEPENDENT P_Sleep_Latency

/METHOD=ENTER Sex Age Time_covid.

EXECUTE.

REGRESSION

/MISSING LISTWISE

/STATISTICS COEFF OUTS R ANOVA

/CRITERIA=PIN(.05) POUT(.10)

/NOORIGIN

/DEPENDENT P_Sleep_Duration

/METHOD=ENTER Sex Age Time_covid.

EXECUTE.

REGRESSION

/MISSING LISTWISE

/STATISTICS COEFF OUTS R ANOVA

/CRITERIA=PIN(.05) POUT(.10)

/NOORIGIN

/DEPENDENT P_Habitual_Sleep_Efficiency

/METHOD=ENTER Sex Age Time_covid.

EXECUTE.

REGRESSION

/MISSING LISTWISE

/STATISTICS COEFF OUTS R ANOVA

/CRITERIA=PIN(.05) POUT(.10)

/NOORIGIN

/DEPENDENT P_Sleep_Disturbances

/METHOD=ENTER Sex Age Time_covid.

EXECUTE.

REGRESSION

/MISSING LISTWISE

/STATISTICS COEFF OUTS R ANOVA

/CRITERIA=PIN(.05) POUT(.10)

/NOORIGIN

/DEPENDENT P_Use_Sleeping_Medication

/METHOD=ENTER Sex Age Time_covid.

EXECUTE.

REGRESSION

/MISSING LISTWISE

/STATISTICS COEFF OUTS R ANOVA

/CRITERIA=PIN(.05) POUT(.10)

/NOORIGIN

/DEPENDENT P_Daytime_dysfunction

/METHOD=ENTER Sex Age Time_covid.

EXECUTE.

REGRESSION

/MISSING LISTWISE

/STATISTICS COEFF OUTS R ANOVA

/CRITERIA=PIN(.05) POUT(.10)

/NOORIGIN

/DEPENDENT MBI_Emotional_exhaustion

/METHOD=ENTER Sex Age Time_covid.

EXECUTE.

REGRESSION

/MISSING LISTWISE

/STATISTICS COEFF OUTS R ANOVA

/CRITERIA=PIN(.05) POUT(.10)

/NOORIGIN

/DEPENDENT MBI_Depersonalization

/METHOD=ENTER Sex Age Time_covid.

EXECUTE.

REGRESSION

/MISSING LISTWISE

/STATISTICS COEFF OUTS R ANOVA

/CRITERIA=PIN(.05) POUT(.10)

/NOORIGIN

/DEPENDENT MBI_Personal_Accomplishment

/METHOD=ENTER Sex Age Time_covid.

EXECUTE.
