# Supplementary material for: Early Psychiatric Impact of COVID-19 Pandemic on the General Population and Healthcare Workers in Italy: A Preliminary Study
Source: Front Psychiatry. 2020 Dec 22;11:561345. doi: 10.3389/fpsyt.2020.561345 (PMC7783153; doi:10.3389/fpsyt.2020.561345)
Supplement: Supplementary file 2 [file Data_Sheet_2.docx]

**Supplementary Material 2**

| 2a. Overall sample | | | | | | |
| --- | --- | --- | --- | --- | --- | --- |
|  | | **Normal** | **Mild** | **Moderate** | **Severe** | **Extremely Severe** |
| DASS-21 Stress | **Overall** | 288  (66.7%) | 55  (12.7%) | 58  (13.4%) | 22  (5.1%) | 9  (2.1%) |
|  | **GP** | 224  (72.5%) | 36  (11.7%) | 35  (11.3%) | 9  (2.9%) | 5  (1.6%) |
|  | **HW** | 64  (52%) | 19  (15.4%) | 23  (18.7%) | 13  (10.6%) | 4  (3.3%) |
| DASS-21 Anxiety | **Overall** | 322  (74.5%) | 52  (12%) | 30  (6.9%) | 11  (2.5%) | 17  (3.9%) |
|  | **GP** | 246  (79.6%) | 32  (10.4%) | 17  (5.5%) | 5  (1.6%) | 9  (2.9%) |
|  | **HW** | 76  (61.8%) | 20  (16.3%) | 13  (10.6%) | 6  (4.9%) | 8  (6.5%) |
| DASS-21 Depression | **Overall** | 277  (64.1%) | 75  (17.4%) | 58  (13.4%) | 12  (2.8%) | 10  (2.3%) |
|  | **GP** | 205  (66.3%) | 50  (16.2%) | 38  (12.3%) | 9  (2.9%) | 7  (2.3%) |
|  | **HW** | 72  (58.5%) | 25  (20.3%) | 20  (16.3%) | 3  (2.4%) | 3  (2.4%) |
|  |  |  |  |  |  |  |
|  | | **Normal** | **Mild** | **Moderate** | **Severe** | |
| IES-R Total Score | **Overall** | 310  (71.8%) | 62  (14.4%) | 15  (3.5%) | 45  (10.4%) | |
|  | **GP** | 232  (75.1%) | 40  (12.9%) | 7  (2.3%) | 30  (9.7%) | |
|  | **HW** | 78  (63.4%) | 22  (17.9%) | 8  (6.5%) | 15  (12.2%) | |
|  |  |  |  |  |  |  |
|  | | **Good Sleepers** | | **Bad Sleepers** | | |
| PSQI Total Score | **Overall** | 183  (42.4%) | | 249  (57.6%) | | |
|  | **GP** | 148  (47.9%) | | 161  (52.1%) | | |
|  | **HW** | 35  (28.5%) | | 88  (71.5%) | | |
|  | | **Low** | **Moderate** | | **High** | |
| MBI Emotional Exhaustion | **HW** | 44  (35.8%) | 23  (18.7%) | | 47  (38.2%) | |
| MBI Depersonalization | **HW** | 22  (17.9%) | 43  (35%) | | 49  (39.8%) | |
|  | | **High** | **Moderate** | | **Low** | |
| MBI Personal Accomplishment | **HW** | 23  (18.7%) | 32  (26%) | | 59  (48%) | |

| 2b. Healthcare workers | | | | | | |
| --- | --- | --- | --- | --- | --- | --- |
|  | | **Normal** | **Mild** | **Moderate** | **Severe** | **Extremely Severe** |
| DASS-21 Stress | **CHW** | 21  (42.9%) | 8  (16.3%) | 12  (24.5%) | 7  (14.3%) | 1  (2%) |
|  | **NCHW** | 43  (58.1%) | 11  (14.9%) | 11  (14.9%) | 6  (8.1%) | 3  (4.1%) |
| DASS-21 Anxiety | **CHW** | 26  (53.1%) | 10  (20.4%) | 8  (16.3%) | 2  (4.1%) | 3  (6.1%) |
|  | **NCHW** | 50  (67.6%) | 10  (13.5%) | 5  (6.8%) | 4  (5.4%) | 5  (6.8%) |
| DASS-21 Depression | **CHW** | 24  (49%) | 17  (34.7%) | 6  (12.2%) | 1  (2%) | 1  (2%) |
|  | **NCHW** | 48  (64.9%) | 8  (10.8%) | 14  (18.9%) | 2  (2.7%) | 2  (2.7%) |
|  | | **Normal** | **Mild** | **Moderate** | **Severe** | |
| IES-R Total Score | **CHW** | 28  (57.1%) | 10  (20.4%) | 4  (8.2%) | 7  (14.3%) | |
|  | **NCHW** | 50  (67.6%) | 12  (16.2%) | 4  (5.4%) | 8  (10.8%) | |
|  | | **Good Sleepers** | | **Bad Sleepers** | | |
| PSQI Total Score | **CHW** | 14  (28.6%) | | 35  (71.4%) | | |
|  | **NCHW** | 21  (28.4%) | | 53  (71.6%) | | |
|  | | **Low** | **Moderate** | | **High** | |
| MBI Emotional Exhaustion | **CHW** | 16  (32.7%) | 5  (10.2%) | | 28  (57.1%) | |
|  | **NCHW** | 28  (43.1%) | 18  (27.7%) | | 19  (29.2%) | |
| MBI Depersonalization | **CHW** | 6  (12.2%) | 19  (38.8%) | | 24  (49%) | |
|  | **NCHW** | 16  (24.6%) | 24  (36.9%) | | 25  (38.5%) | |
|  | | **High** | **Moderate** | | **Low** | |
| MBI Personal Accomplishment | **CHW** | 14  (28.6%) | 14  (28.6%) | | 21  (42.9%) | |
|  | **NCHW** | 9  (13.8%) | 18  (27.7%) | | 38  (58.5%) | |

Supplementary Material 1a: Psychopathological assessment in the overall sample: participants distribution on severity scales.

Supplementary Material 1b: Psychopathological assessment in health care workers only: participants distribution on severity scales.

Abbreviations: CHW: healthcare workers directly in contact with COVID-19 patients; DASS-21: Depression, Anxiety and Stress Scale – 21 items; GP = General Public; HW: healthcare Workers; IES-R: Impact of Event Scale-Revised; MBI: Maslach Burnout Inventory; N/A: Not Applicable; NCHW: healthcare workers not directly in contact with COVID-19 patients; PSQI: Pittsburgh Sleep Quality Index
