# Supplementary material for: Early Psychiatric Impact of COVID-19 Pandemic on the General Population and Healthcare Workers in Italy: A Preliminary Study
Source: Front Psychiatry. 2020 Dec 22;11:561345. doi: 10.3389/fpsyt.2020.561345 (PMC7783153; doi:10.3389/fpsyt.2020.561345)
Supplement: Supplementary file 3 [file Data_Sheet_3.docx]

**Supplementary Material 3**

| 3a. Whole Sample | | | | | |
| --- | --- | --- | --- | --- | --- |
|  |  | b | t | p | R2 |
| DASS-21 Total score | Intercept | 15.7 | 5.2 | <0.001 | 0.07 |
|  | Gender | **2.2** | **2.3** | **0.023** |  |
|  | Age | **-0.1** | **-3.2** | **0.002** |  |
|  | Years of education | -0.1 | -0.6 | 0.551 |  |
|  | Healthcare workers | **3.8** | **3.8** | **<0.001** |  |
| DASS-21 Stress | Intercept | 6.4 | 5.0 | 0.000 | 0.093 |
|  | Gender | **0.9** | **2.2** | **0.030** |  |
|  | Age | **-0.1** | **-3.9** | **<0.001** |  |
|  | Years of education | 0.1 | 0.8 | 0.419 |  |
|  | Healthcare workers | **1.8** | **4.1** | **<0.001** |  |
|  |  |  |  |  |  |
| DASS-21 Anxiety | Intercept | 3809.0 | 3781.0 | <0.001 | 0.078 |
|  | Gender | **0.956** | **2948.0** | **0.003** |  |
|  | Age | **- 0.029** | **- 2.39** | **0.017** |  |
|  | Years of education | -0.088 | -1.602 | 0.110 |  |
|  | Healthcare workers | **1444.0** | **4238.0** | **< 0.001** |  |
| DASS-21 Depression | Intercept | 5.5 | 4679.0 | <0.001 | 0.018 |
|  | Gender | 0.4 | 0.991 | 0.322 |  |
|  | Age | 0.0 | -1.837 | 0.067 |  |
|  | Years of education | -0.1 | -1.033 | 0.302 |  |
|  | Healthcare workers | 0.6 | 1569.0 | 0.117 |  |
| IES-R Total score | Intercept | 15.4 | 3.6 | <0.001 | 0.05 |
|  | Gender | **4.9** | **3.5** | **<0.001** |  |
|  | Age | -0.1 | -1.6 | 0.120 |  |
|  | Years of education | 0.1 | 0.4 | 0.680 |  |
|  | Healthcare workers | **3.1** | **2.2** | **0.032** |  |
| IES-R Avoidance | Intercept | 1.0 | 4.8 | 0.000 | 0.027 |
|  | Gender | **0.1** | **2.2** | **0.032** |  |
|  | Age | **0.0** | **-2.6** | **0.010** |  |
|  | Years of education | 0.0 | -0.1 | 0.925 |  |
|  | Healthcare workers | 0.0 | 0.3 | 0.767 |  |
| IES-R Intrusion | Intercept | 0.4 | 1.9 | 0.064 | 0.075 |
|  | Gender | **0.3** | **3.9** | **<0.001** |  |
|  | Age | 0.0 | 0.3 | 0.769 |  |
|  | Years of education | 0.0 | 0.6 | 0.577 |  |
|  | Healthcare workers | **0.3** | **3.6** | **<0.001** |  |
| IES-R Hyperarousal | Intercept | 0.7 | 3.2 | 0.002 | 0.05 |
|  | Gender | **0.2** | **3.4** | **0.001** |  |
|  | Age | **0.0** | **-2.3** | **0.025** |  |
|  | Years of education | 0.0 | 0.7 | 0.494 |  |
|  | Health worker | 0.1 | 1.7 | 0.090 |  |
| PSQI Total Score | Intercept | 3.5 | 3.6 | <0.001 | 0.084 |
|  | Gender | 0.6 | 1.9 | 0.058 |  |
|  | Age | **0.0** | **3.2** | **0.002** |  |
|  | Years of education | 0.0 | -0.2 | 0.838 |  |
|  | Healthcare workers | **1.6** | **4.7** | **<0.001** |  |
| PSQI – Subjective Sleep Quality | Intercept | 0.6 | 3.0 | 0.003 | 0.055 |
|  | Gender | **0.2** | **3.0** | **0.003** |  |
|  | Age | 0.0 | 0.9 | 0.359 |  |
|  | Years of education | 0.0 | 1.0 | 0.327 |  |
|  | Healthcare workers | **0.2** | **3.0** | **0.003** |  |
| PSQI – Sleep Latency | Intercept | 1.3 | 4.5 | <0.001 | 0.029 |
|  | Gender | 0.2 | 2.3 | 0.020 |  |
|  | Age | 0.0 | -1.4 | 0.154 |  |
|  | Years of education | 0.0 | -1.2 | 0.234 |  |
|  | Healthcare workers | 0.2 | 1.8 | 0.075 |  |
| PSQI – Sleep Duration | Intercept | 0.3 | 1.1 | 0.255 | 0.133 |
|  | Gender | -0.1 | -1.5 | 0.144 |  |
|  | Age | **0.0** | **4.7** | **<0.001** |  |
|  | Years of education | 0.0 | -0.3 | 0.748 |  |
|  | Healthcare workers | **0.6** | **6.3** | **<0.001** |  |
| PSQI – Habitual Sleep Efficiency | Intercept | 0.2 | 0.7 | 0.470 | 0.035 |
|  | Gender | 0.1 | 1.5 | 0.143 |  |
|  | Age | 0.0 | 1.9 | 0.054 |  |
|  | Years of education | 0.0 | -0.4 | 0.696 |  |
|  | Healthcare workers | **0.3** | **2.9** | **0.004** |  |
| PSQI – Sleep Disturbances | Intercept | 0.9 | 5.9 | <0.001 | 0.030 |
|  | Gender | **0.2** | **3.2** | **0.001** |  |
|  | Age | 0.0 | 1.1 | 0.263 |  |
|  | Years of education | 0.0 | -0.8 | 0.397 |  |
|  | Healthcare workers | 0.1 | 1.0 | 0.330 |  |
| PSQI – Use of Sleeping Medications | Intercept | -0.3 | -1.3 | 0.207 | 0.054 |
|  | Gender | -0.1 | -0.8 | 0.438 |  |
|  | Age | **0.0** | **4.5** | **<0.001** |  |
|  | Years of education | 0.0 | 0.7 | 0.511 |  |
|  | Healthcare workers | 0.1 | 1.5 | 0.141 |  |
| PSQI – Daytime Dysfunction | Intercept | 0.5 | 2.5 | 0.013 | 0.028 |
|  | Gender | 0.1 | 1.7 | 0.094 |  |
|  | Age | 0.0 | 1.2 | 0.244 |  |
|  | Years of education | 0.0 | 0.6 | 0.566 |  |
|  | Healthcare workers | **0.1** | **2.3** | **0.020** |  |

| 3b. Healthcare Workers | | | | | |
| --- | --- | --- | --- | --- | --- |
|  |  | b | t | p | R2 |
| DASS-21 Total score | Intercept | 13312.0 | 3605.0 | < 0.001 | 0.065 |
|  | **Gender** | **5.3** | **2.7** | **0.008** |  |
|  | Age | -0.1 | -0.9 | 0.396 |  |
|  | COVID-19 | 1.5 | 0.9 | 0.387 |  |
| DASS-21 Stress | Intercept | 6713.0 | 4195.0 | < 0.001 | 0.073 |
|  | **Gender** | **2.3** | **2.7** | **0.008** |  |
|  | Age | 0.0 | -0.9 | 0.365 |  |
|  | COVID-19 | 1.0 | 1.3 | 0.188 |  |
| DASS-21 Anxiety | Intercept | 3002.0 | 2362.0 | 0.020 | 0.104 |
|  | **Gender** | **2.3** | **3.4** | **0.001** |  |
|  | Age | 0.0 | -1.5 | 0.148 |  |
|  | COVID-19 | 0.3 | 0.6 | 0.548 |  |
| DASS-21 Depression | Intercept | 3.6 | 2.7 | 0.009 | 0.007 |
|  | Gender | 0.6 | 0.9 | 0.370 |  |
|  | Age | 0.0 | 0.1 | 0.905 |  |
|  | COVID-19 | 0.1 | 0.2 | 0.812 |  |
| IES-R Total score | Intercept | 15.4 | 2.7 | 0.008 | 0.051 |
|  | **Gender** | **6.9** | **2.3** | **0.024** |  |
|  | Age | 0.0 | -0.2 | 0.847 |  |
|  | COVID-19 | 3.2 | 1.2 | 0.219 |  |
| IES-R Avoidance | Intercept | 0.8 | 3.1 | 0.002 | 0.02 |
|  | Gender | 0.2 | 1.4 | 0.155 |  |
|  | Age | 0.0 | -0.6 | 0.565 |  |
|  | COVID-19 | 0.0 | 0.2 | 0.803 |  |
| IES-R Intrusion | Intercept | 0.7 | 2.2 | 0.029 | 0.075 |
|  | **Gender** | **0.3** | **2.2** | **0.033** |  |
|  | Age | 0.0 | 0.0 | 0.989 |  |
|  | **COVID-19** | **0.3** | **2.3** | **0.021** |  |
| IES-R Hyperarousal | Intercept | 0.6 | 1.9 | 0.058 | 0.055 |
|  | **Gender** | **0.4** | **2.6** | **0.010** |  |
|  | Age | 0.0 | 0.0 | 0.973 |  |
|  | COVID-19 | 0.1 | 0.5 | 0.637 |  |
| PSQI Total Score | Intercept | 2.1 | 1.5 | 0.141 | 0.083 |
|  | Gender | 1.0 | 1.3 | 0.193 |  |
|  | **Age** | **0.1** | **2.9** | **0.004** |  |
|  | COVID-19 | 0.4 | 0.6 | 0.531 |  |
| PSQI – Subjective Sleep Quality | Intercept | 0.4 | 1.6 | 0.118 | 0.092 |
|  | **Gender** | **0.4** | **2.5** | **0.014** |  |
|  | Age | 0.0 | 2.3 | 0.026 |  |
|  | COVID-19 | 0.1 | 0.9 | 0.397 |  |
| PSQI – Sleep Latency | Intercept | 0.9 | 2.2 | 0.027 | 0.004 |
|  | Gender | 0.1 | 0.6 | 0.529 |  |
|  | Age | 0.0 | 0.3 | 0.742 |  |
|  | COVID-19 | 0.0 | 0.1 | 0.957 |  |
| PSQI – Sleep Duration | Intercept | 0.3 | 0.6 | 0.519 | 0.071 |
|  | Gender | 0.0 | 0.2 | 0.846 |  |
|  | **Age** | **0.0** | **2.9** | **0.004** |  |
|  | COVID-19 | 0.1 | 0.7 | 0.507 |  |
| PSQI – Habitual Sleep Efficiency | Intercept | -0.2 | -0.6 | 0.526 | 0.082 |
|  | Gender | 0.0 | -0.2 | 0.873 |  |
|  | **Age** | **0.0** | **2.3** | **0.021** |  |
|  | COVID-19 | 0.4 | 2.2 | 0.032 |  |
| PSQI – Sleep Disturbances | Intercept | 0.6 | 3.0 | 0.004 | 0.056 |
|  | **Gender** | **0.3** | **2.3** | **0.023** |  |
|  | Age | 0.0 | 1.2 | 0.224 |  |
|  | COVID-19 | 0.0 | 0.5 | 0.605 |  |
| PSQI – Use of Sleeping Medications | Intercept | -0.4 | -1.1 | 0.287 | 0.076 |
|  | Gender | 0.0 | 0.2 | 0.867 |  |
|  | **Age** | **0.0** | **2.8** | **0.006** |  |
|  | COVID-19 | -0.2 | -1.5 | 0.142 |  |
| PSQI – Daytime Dysfunction | Intercept | 0.5 | 2.2 | 0.027 | 0.036 |
|  | Gender | 0.2 | 1.9 | 0.059 |  |
|  | Age | 0.0 | 0.9 | 0.386 |  |
|  | COVID-19 | 0.0 | 0.2 | 0.844 |  |
| MBI Emotional Exhaustion | Intercept | 19.4 | 4.1 | <0.001 | 0.120 |
|  | **Gender** | **5.1** | **2.0** | **0.044** |  |
|  | Age | 0.0 | -0.4 | 0.717 |  |
|  | **COVID-19** | **7.2** | **3.3** | **0.001** |  |
| MBI Depersonalization | Intercept | 11.3 | 5.2 | <0.001 | 0.032 |
|  | Gender | -0.4 | -0.3 | 0.757 |  |
|  | Age | 0.0 | -0.8 | 0.443 |  |
|  | COVID-19 | 1.7 | 1.7 | 0.086 |  |
| MBI Personal Accomplishment | Intercept | 30.0 | 7.6 | <0.001 | 0.032 |
|  | Gender | -0.4 | -0.2 | 0.840 |  |
|  | Age | 0.0 | 0.3 | 0.745 |  |
|  | COVID-19 | 3.3 | 1.9 | 0.065 |  |

| 3c. COVID-19 Healthcare Workers | | | | | |
| --- | --- | --- | --- | --- | --- |
|  |  | b | t | p | R2 |
| DASS-21 Total score | Intercept | 16.5 | 3.0 | 0.004 | 0.081 |
|  | Gender | 4.2 | 1.7 | 0.100 |  |
|  | Age | -0.1 | -1.0 | 0.323 |  |
|  | Time COVID-19 | 0.1 | 0.5 | 0.623 |  |
| DASS-21 Stress | Intercept | 9.3 | 3.6 | 0.001 | 0.107 |
|  | Gender | 2.1 | 1.7 | 0.087 |  |
|  | Age | -0.1 | -1.5 | 0.153 |  |
|  | Time COVID-19 | 0.0 | 0.6 | 0.564 |  |
| DASS-21 Anxiety | Intercept | 2.4 | 1.2 | 0.219 | 0.109 |
|  | **Gender** | **2.4** | **2.7** | **0.010** |  |
|  | Age | 0.0 | -0.9 | 0.359 |  |
|  | Time COVID-19 | 0.0 | 1.1 | 0.266 |  |
| DASS-21 Depression | Intercept | 4.9 | 2.3 | 0.023 | 0.005 |
|  | Gender | -0.2 | -0.2 | 0.834 |  |
|  | Age | 0.0 | 0.0 | 0.997 |  |
|  | Time COVID-19 | 0.0 | -0.4 | 0.657 |  |
| IES-R Total score | Intercept | 17.0 | 1.7 | 0.095 | 0.055 |
|  | Gender | 6.0 | 1.3 | 0.195 |  |
|  | Age | -0.1 | -0.3 | 0.760 |  |
|  | Time COVID-19 | 0.2 | 1.0 | 0.314 |  |
| IES-R Avoidance | Intercept | 0.6 | 1.5 | 0.146 | 0.035 |
|  | Gender | 0.2 | 1.1 | 0.280 |  |
|  | Age | 0.0 | -0.1 | 0.884 |  |
|  | Time COVID-19 | 0.0 | 0.8 | 0.453 |  |
| IES-R Intrusion | Intercept | 1.1 | 1.9 | 0.064 | 0.045 |
|  | Gender | 0.3 | 1.0 | 0.321 |  |
|  | Age | 0.0 | -0.5 | 0.622 |  |
|  | Time COVID-19 | 0.0 | 1.1 | 0.291 |  |
| IES-R Hyperarousal | Intercept | 0.6 | 1.1 | 0.262 | 0.067 |
|  | Gender | 0.4 | 1.6 | 0.115 |  |
|  | Age | 0.0 | -0.1 | 0.913 |  |
|  | Time COVID-19 | 0.0 | 0.9 | 0.366 |  |
| PSQI Total Score | Intercept | 4.5 | 1.9 | 0.068 | 0.065 |
|  | Gender | 0.6 | 0.5 | 0.609 |  |
|  | Age | 0.0 | 0.3 | 0.794 |  |
|  | Time COVID-19 | 0.1 | 1.6 | 0.111 |  |
| PSQI – Subjective Sleep Quality | Intercept | 0.9 | 1.9 | 0.064 | 0.053 |
|  | Gender | 0.2 | 0.8 | 0.410 |  |
|  | Age | 0.0 | 0.1 | 0.882 |  |
|  | Time COVID-19 | 0.0 | 1.4 | 0.179 |  |
| PSQI – Sleep Latency | Intercept | 1.0 | 1.4 | 0.155 | 0.031 |
|  | Gender | 0.1 | 0.3 | 0.751 |  |
|  | Age | 0.0 | -0.4 | 0.699 |  |
|  | Time COVID-19 | 0.0 | 1.2 | 0.254 |  |
| PSQI – Sleep Duration | Intercept | 1.4 | 1.9 | 0.066 | 0.008 |
|  | Gender | 0.0 | -0.1 | 0.919 |  |
|  | Age | 0.0 | -0.1 | 0.903 |  |
|  | Time COVID-19 | 0.0 | 0.6 | 0.563 |  |
| PSQI – Habitual Sleep Efficiency | Intercept | 0.5 | 0.7 | 0.474 | 0.051 |
|  | Gender | 0.1 | 0.4 | 0.707 |  |
|  | Age | 0.0 | -0.2 | 0.842 |  |
|  | Time COVID-19 | 0.0 | 1.5 | 0.132 |  |
| PSQI – Sleep Disturbances | Intercept | 0.6 | 1.8 | 0.081 | 0.178 |
|  | Gender | 0.2 | 1.3 | 0.193 |  |
|  | Age | 0.0 | -0.4 | 0.674 |  |
|  | **Time COVID-19** | **0.0** | **2.9** | **0.005** |  |
| PSQI – Use of Sleeping Medications | Intercept | -1.1 | -2.6 | 0.013 | 0.26 |
|  | Gender | 0.0 | 0.2 | 0.843 |  |
|  | **Age** | **0.0** | **4.0** | **<0.001** |  |
|  | Time COVID-19 | 0.0 | -0.8 | 0.414 |  |
| PSQI – Daytime Dysfunction | Intercept | 1.1 | 3.8 | <0.001 | 0.044 |
|  | Gender | 0.0 | -0.2 | 0.816 |  |
|  | Age | 0.0 | -1.3 | 0.205 |  |
|  | Time COVID-19 | 0.0 | 0.8 | 0.412 |  |
| MBI Emotional Exhaustion | Intercept | 21.1 | 2.4 | 0.023 | 0.107 |
|  | Gender | 7.6 | 1.8 | 0.072 |  |
|  | Age | -0.1 | -0.4 | 0.688 |  |
|  | Time COVID-19 | 0.3 | 1.5 | 0.134 |  |
| MBI Depersonalization | Intercept | 14.5 | 3.7 | 0.001 | 0.027 |
|  | Gender | 0.1 | 0.0 | 0.964 |  |
|  | Age | -0.1 | -1.1 | 0.276 |  |
|  | Time COVID-19 | 0.0 | 0.3 | 0.731 |  |
| MBI Personal Accomplishment | Intercept | 34.8 | 5.6 | <0.001 | 0.04 |
|  | Gender | -3.6 | -1.2 | 0.218 |  |
|  | Age | 0.0 | 0.2 | 0.823 |  |
|  | Time COVID-19 | 0.0 | 0.3 | 0.743 |  |

Supplementary Material 3a: Regression analysis in the whole sample;

Supplementary Material 3b: Regression analysis in a group of healthcare workers only;

Supplementary Material 3c: Regression analysis in a group of healthcare workers directly in contact with COVID-19 patients only.

Abbreviations: DASS-21: Depression, Anxiety and Stress Scale – 21 items; IES-R: Impact of Event Scale-Revised; MBI: Maslach Burnout Inventory; N/A: Not Applicable; PSQI: Pittsburgh Sleep Quality Index
